# Assessing disease activity of rheumatoid arthritis patients and drug-utilization patterns of biologic disease-modifying antirheumatic drugs in the Tuscany region, Italy

**DOI:** 10.3389/fphar.2023.1244486

**Published:** 2023-09-25

**Authors:** Irma Convertino, Massimiliano Cazzato, Silvia Tillati, Sabrina Giometto, Rosa Gini, Giulia Valdiserra, Emiliano Cappello, Sara Ferraro, Marco Bonaso, Claudia Bartolini, Olga Paoletti, Valentina Lorenzoni, Leopoldo Trieste, Matteo Filippi, Giuseppe Turchetti, Michele Cristofano, Corrado Blandizzi, Marta Mosca, Ersilia Lucenteforte, Marco Tuccori

**Affiliations:** ^1^ Unit of Pharmacology and Pharmacovigilance, Department of Clinical and Experimental Medicine, University of Pisa, Pisa, Italy; ^2^ Unit of Rheumatology, University Hospital of Pisa, Pisa, Italy; ^3^ Unit of Medical Statistics, Department of Clinical and Experimental Medicine, University of Pisa, Pisa, Italy; ^4^ Tuscan Regional Healthcare Agency, Florence, Italy; ^5^ Institute of Management, Scuola Superiore Sant’Anna, Pisa, Italy; ^6^ Direzione Medica di Presidio, University Hospital of Pisa, Pisa, Italy; ^7^ Unit of Adverse Drug Reactions Monitoring, University Hospital of Pisa, Pisa, Italy

**Keywords:** DAS28, initiation, discontinuation, biologic, DMARD, real-world, drug-utilization

## Abstract

**Introduction:** The disease activity associated with the drug-utilization patterns of biologic Disease Modifying Anti-Rheumatic Drugs (DMARDs) is poorly investigated in real-world studies on rheumatoid arthritis (RA) patients. To investigate the relationship between biologic DMARD initiation/discontinuations in RA patients identified in the healthcare administrative databases of Tuscany and the Disease Activity Score 28 (DAS28) reported in the medical charts.

**Methods:** This retrospective population-based study included RA’s first-ever biologic DMARD users of the Pisa University Hospital from 2014 to 2016. Patients were followed up until 31 December 2019. We evaluated the DAS28 recorded before (T0) and after (T1) the biologic DMARD initiation and before (TD0) and after (TD1) discontinuations. Patients were classified as “off-target” (DAS28 > 3.2) or “in-target” (DAS28 ≤ 3.2). We described the disease activity trends at initiation and discontinuation.

**Results:** Ninety-five users were included (73 women, mean age 59.6). Among 70 patients (74%) with at least three DAS28 measures, 28 (40.0%) were off-target at T0 and 38 (54.3%) in-target at T1. Thirty-three (47%) patients had at least one discontinuation, among those with at least three DAS28 assessments. In the disease activity trend, disease stability or improvement was observed in 28 out of 37 (75.7%) patients at initiation and in 24 out of 37 (64.9%) at discontinuation.

**Discussion:** Biologic DMARD discontinuations identified in the healthcare administrative databasese of Tuscany are frequently observed in situations of controlled RA disease. Further studies are warranted to confirm that these events can be used in studies using healthcare administrative databases as proxies of treatment effectiveness.

## 1 Introduction

Healthcare administrative databases (HADs) are demonstrated to be reliable data sources for drug utilization studies (Gini et al., 2014; Trifirò et al., 2021). However, investigating clinical outcomes associated with drug use in these databases is conditioned by the aims these data are collected for, particularly the management of healthcare costs. Therefore, proxies must often define clinical outcomes (Convertino et al., 2021a). For example, the treatment initiation identified in these databases can be interpreted as a clinical need (i.e., an uncontrolled disease) and a change in therapy (switching) or interruption (discontinuation) as efficacy loss or as a consequence of tolerability problems.

The Pathfinder project (EUPAS29263) (Convertino et al., 2021d) was developed to describe the use of biological drugs in rheumatoid arthritis (RA) patients and the related clinical outcomes by combining data from the HAD of the Tuscan region with the information contained in the individual medical charts. The extraction algorithm of subjects with RA demonstrated high values of sensitivity (0.93; 95% confidence interval, CI, 0.86–0.97), specificity (0.84; 95% CI 0.78–0.90), and positive predictive value (0.78; 95% CI 0.70–0.85) (Convertino et al., 2021b). The project characterized the use of biological drugs in these patients, classifying them based on adherence trajectories. This evaluation observed that about 88% of the subjects fall into the adherence category of continuous users characterized by alternation of phases of treatment coverage with phases not covered by treatment (Convertino et al., 2021c). In accordance with the guidelines that provide for the biologic prescription in subjects with uncontrolled disease, and the treatment tapering in subjects showing disease remission (Smolen et al., 2010; Smolen et al., 2014; Smolen et al., 2017), we hypothesized that the disease activity is high in correspondence with events of biologic initiation and improves or remains stable in subsequent assessments. Disease activity is also expected to be reduced before discontinuations and to remain almost stable thereafter.

To test these hypotheses, we described the disease activity reported in the medical charts of an RA population in correspondence with the events of initiation and discontinuations of biologic disease-modifying antirheumatic drugs (DMARDs), identified from the HAD of the Tuscany Region (Italy).

## 2 Materials and methods

### 2.1 Study design and data sources

This investigation is part of the Pathfinder (Convertino et al., 2021d), a retrospective population-based cohort study on RA first ever biologic DMARD users extracted from the HAD of Tuscany. The regional healthcare system, comprising the national, universal, single-payer, and public health system, provides healthcare assistance to over 3 million Tuscan residents. All data about services supplied have been recorded electronically in the HAD since 2004. Data are periodically analyzed by the Agenzia Regionale di Sanità Toscana (ARST), and these have been used to conduct pharmacoepidemiological studies (Gini et al., 2014; Trifirò et al., 2021). The study was conducted from 1 January 2004 to 31 December 2019. We extracted data on 29 April 2020 from the HAD. We also collected information from the corresponding medical charts of the Rheumatology Unit of Pisa University Hospital. We used the following repositories encompassed in the Tuscan HAD: drug supply to inpatient and outpatient databases (Anatomical Therapeutic Chemical, ATC, Classification codes), exemptions from the co-payment database (exemption from co-payment codes), hospital discharge records, Emergency Department (ED) admission records (International Classification of Diseases Ninth Revision, ICD-9, codes), and outpatient services for specialist visits. The pseudo-anonymized information of Tuscan RA first ever biologic DMARD users identified by a validated algorithm (Convertino et al., 2021b) from the HAD was linked to the corresponding medical chart data in the Rheumatology Ward of Pisa University Hospital. According to the ethical and data protection requirements, data were managed by the Hospital Healthcare Office and through a unique identification number (Supplementary Material S1, Supplementary Figure S1). The informed consent for participating in the study was obtained from patients during the scheduled visits or by phone. From the medical charts, the following information was collected retrospectively: RA diagnosis date, Disease Activity Score 28 (DAS28), adverse events, adverse event dates, and dates of the DAS28 assessments. Information extracted from the HAD involved the biologic DMARD supplies, RA visits, RA diagnosis, RA exemption from co-payment, the first biologic DMARD supply date, the index date (ID), and the dates of the subsequent biologic DMARD dispensations.

The Pathfinder study received consent from the European Network of Centres for Pharmacoepidemiology and Pharmacovigilance (ENCePP) (EUPAS29263) (Convertino et al., 2021d) and was approved by the Ethical Committee of Pisa University Hospital (Protocol number 18724). This article was written in accordance with the guidelines on conducting and reporting drug utilization studies (Vrijens et al., 2012; De Geest et al., 2018; Dima et al., 2021).

### 2.2 Study population

During the validation analysis (Convertino et al., 2021b), we identified RA first ever biologic DMARD (infliximab, adalimumab, certolizumab pegol, etanercept, golimumab, abatacept, tocilizumab, sarilumab, and rituximab) users in the period between 1 January 2014 and 31 December 2016 (inclusion period), accessing healthcare facilities at the Rheumatology Unit of Pisa University Hospital in the year preceding the ID. First ever users were defined by no biologic DMARD dispensation recorded in the period ranging from the first record available in the Tuscan HAD to the ID (look-back period). We decided to exclude patients with rituximab as an index drug since the timing of administration is different from that of other biologic DMARDs, and this could have affected the frequency of DAS28 assessments. We followed up patients from the ID until the occurrence of the following events: disenrollment from the healthcare coverage plan, death, or end of the study period (31 December 2019), whichever came first.

### 2.3 Measurement

For each patient, we identified the ID and the date of each discontinuation event, defined as the first day not covered with biologic DMARDs. The coverage was calculated based on the defined daily dose (DDD) (WHO, 2021) and the number of doses supplied plus a grace period of 60 days not covered by treatment (i.e., without any other biologic DMARD supply). Disease activity was measured using the DAS28. The disease activity recorded during RA visits was classified according to EULAR guidelines into two clinical categories based on DAS28 values (Fransen and van Riel, 2005): i) in-target disease with DAS28 ≤ 3.2 and ii) off-target disease with DAS28 > 3.2.

**FIGURE 1:**
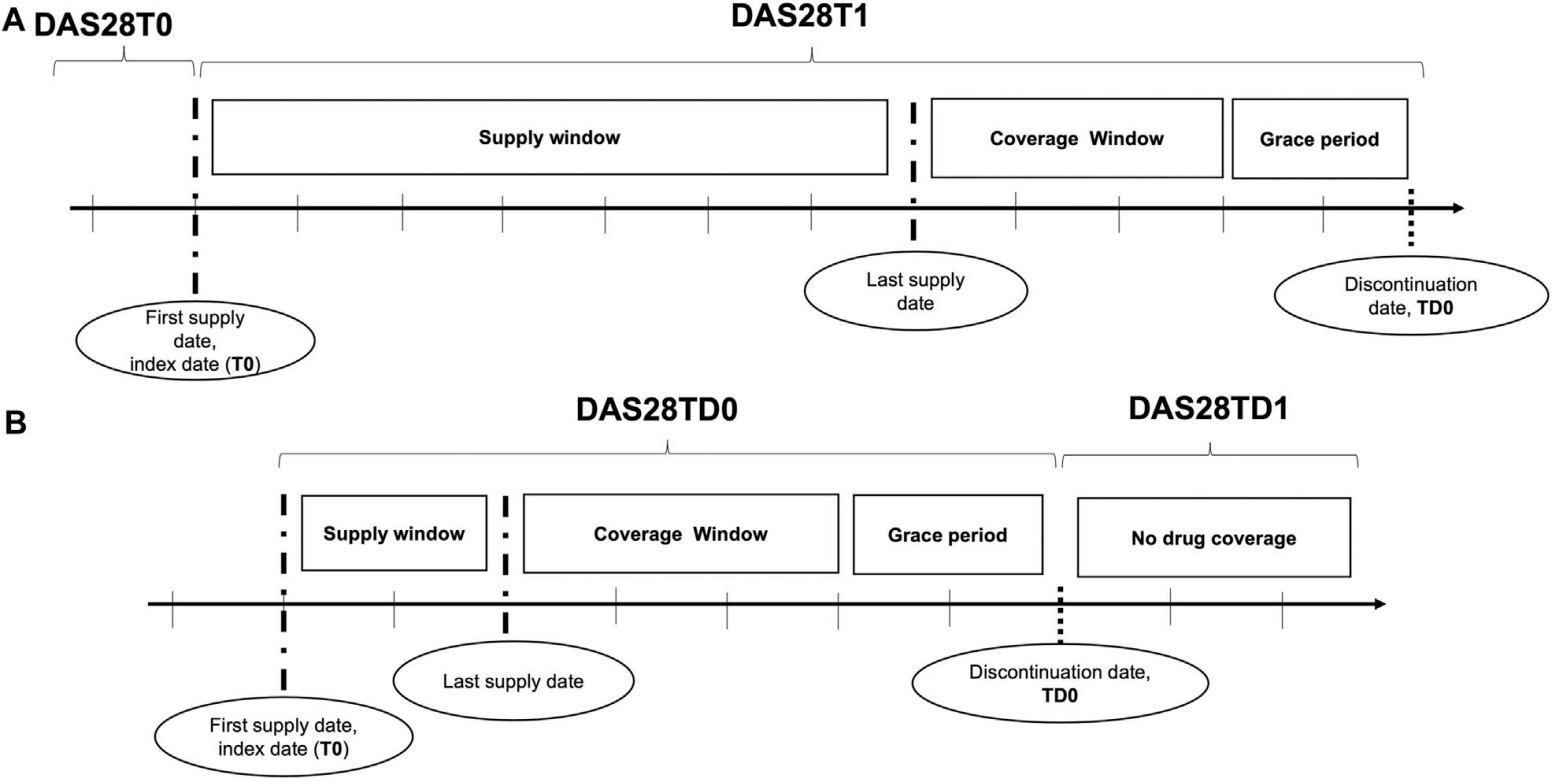
Disease activity assessments: **(A)** at the initiation of biologic DMARDs and **(B)** at the discontinuations. The disease activity was assessed through the DAS28 at initiation of biologic DMARDs **(A)** and at discontinuation **(B)**. Section A displayed how DAS28 was detected at initiation: the DAS28 recorded at the visit before (T0) the first biologic DMARD supply, corresponding to the index date, and that after the index date (T1) were used. Section B showed the DAS28 detection at discontinuations: the DAS28 reported in medical charts at the visit before (TD0) the discontinuation date, defined by the end of the drug coverage plus a grace period, and that after the discontinuation date (TD1) were adopted for the investigation.

We defined the DAS28T0 as the DAS28 value recorded in the closest date before the ID (T0) (including the ID), the DAS28T1 as the DAS28 recorded in the closest date after the ID (T1), the DAS28TD0 as the DAS28 recorded in the closest date before the date of any discontinuation event (TD0) (including the date of the discontinuation event), and the DAS28TD1 as the DAS28 recorded in the closest date after the date of any discontinuation event (TD1) (Figure 1). We measured improvements in DAS28 by classifying the changes according to the EULAR response criteria (Fransen and van Riel, 2005): i) good, when a change >1.2 of the DAS28 from DAS28T0 to DAS28T1 (or from DAS28TD0 to DAS28TD1) was recorded; ii) moderate, when a change > 0.6 of the DAS28 from DAS28T0 to DAS28T1 (or from DAS28TD0 to DAS28TD1) was registered; and iii) no improvement, when the change of DAS28 from T0 to T1 or from TD0 to TD1 ranged between −0.5 and 0.

We considered the following variables as baseline characteristics: time invariant (age at ID and gender), single event (index biologic DMARDs, conventional synthetic DMARDs, azathioprine, cyclophosphamide, ciclosporin, hydroxychloroquine sulfate, leflunomide, methotrexate, mycophenolate mofetil, and sulfasalazine supplied in 3 months prior to ID), and time variant (RA disease duration, as the time from the date of diagnosis in the medical chart to the ID recorded in the HAD, days).

### 2.4 Data analysis

**FIGURE 2:**
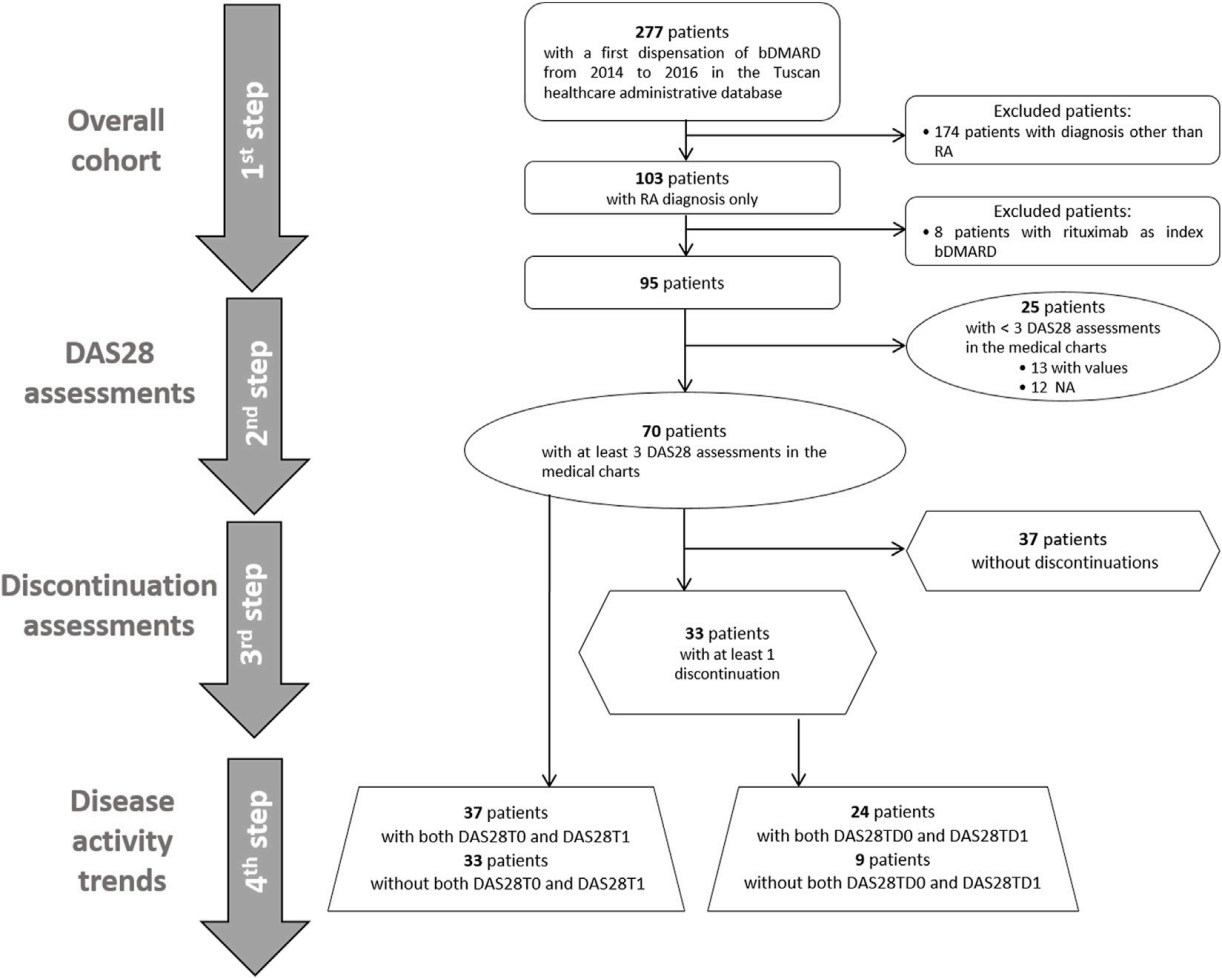
Flow chart of selection of the cohorts for the study analyses. First ever biologic DMARD users from 2014 to 2016 with rheumatoid arthritis were selected, and we included 95 patients in the first-step analysis. In the second step, patients were classified based on the presence of at least three DAS28 assessments reported in the medical charts or not. In the third step, patients were distinguished between those with at least one discontinuation and those without any discontinuation. In the fourth step, the disease activity trend was evaluated at initiation and at discontinuations in available patients with both DAS28T0 and DAS28T1 and DAS28TD0 and DAS28TD1, respectively.

We performed a step-by-step descriptive analysis by progressively classifying the population based on DAS28 measure availability and occurrence of discontinuation events (Figure 2). The results were reported as the numbers and percentages for categorical variables and as the mean and standard deviation (SD) or median and interquartile range [IQR] for continuous variables. In the first step, we described the baseline characteristics of all RA patients. In the second step, we classified patients based on the presence of at least three available DAS28 assessments, and we tested these two groups for differences at the baseline by using the *t*-test and chi-square test, as appropriate. In the group with at least three DAS28 assessments, we described available DAS28T0 and DAS28T1. Discontinuation events were identified, and DAS28TD0 and DAS28TD1 were described. We computed discontinuations as dichotomous variables and categorical variables: no discontinuation, 1 discontinuation, 2–3 discontinuations, and ≥4 discontinuations. In the third step, among patients with at least three DAS28 available, we identified those with at least one discontinuation. We tested the baseline differences between continuers and discontinuers, and we described the related DAS28 observed at T0, T1, TD0, and TD1 and the corresponding time elapsed (days). In the fourth step, we separately analyzed the disease activity trends at the ID and at discontinuations. We restricted the evaluation to patients with records of both DAS28T0 and DAS28T1, and with records of both DAS28TD0 and DAS28TD1 within the group of patients with at least three DAS28 available. We used Sankey plots to illustrate the variation in disease activity with respect to the index date and discontinuation date, where the width of the flows represents the proportion of subjects. To check for discontinuations, possibly due to safety reasons, we retrieved adverse events recorded in patients from medical charts included in the analysis for disease activity trends, and we estimated the time between the date of the event and that of the discontinuation.

We performed a sensitivity analysis by varying the grace period for estimating discontinuations to 30 days. All these analyses were performed on anonymized data using R, version 3.6.3.

## 3 Results

Overall, 95 patients with RA first ever biologic DMARD use had the inclusion criteria (Figure 2). The majority of biologic DMARD first ever users were women (76.8%), and the mean age was 59.6 (SD 12.1). The index biologic DMARDs most frequently supplied were as follows: abatacept (33.7%), etanercept (29.5%), and adalimumab (15.8%). At baseline, 66 patients had at least one supply of csDMARDs, with hydroxychloroquine sulfate (33.7%) and methotrexate (30.5%) as the most frequently observed. The median time elapsed from the RA diagnosis date, and the first biologic DMARD supply was 2,633 [IQR = 966.5–5,518.5] days, i.e., 7.2 years, and most part of the biologic DMARD first ever users belonged to the categories exceeding the 5 years from RA diagnosis (Table 1 and Supplementary Figure S2).

**TABLE 1:** Baseline characteristics of the included RA first ever biologic DMARD users.

	RA first ever biologic DMARD users with DAS28 assessments			*p-value*
	Overall	With at least three DAS28 values	With <3 DAS28 values	
Patients, n (%)	95 (100)	70 (74)	25 (26)	**—**
Females, n (%)	73 (76.8)	55 (78.5)	18 (72)	0.461
Age, mean (SD)	59.6 (12.1)	59.3 (12.4)	60.4 (11.7)	0.708
*Index biologic DMARDs*				0.235
Adalimumab, n (%)	15 (15.8)	11 (15.7)	4 (16.0)	
Certolizumab pegol, n (%)	8 (8.4)	4 (5.7)	4 (16.0)	
Etanercept, n (%)	28 (29.5)	20 (28.6)	8 (32.0)	
Golimumab, n (%)	7 (7.4)	4 (5.7)	3 (12.0)	
Infliximab, n (%)	1 (1.1)	1 (1.4)	-	
Abatacept, n (%)	32 (33.7)	28 (40.0)	4 (16.0)	
Tocilizumab, n (%)	4 (4.2)	2 (2.9)	2 (8.0)	
*csDMARDs*				
At least one csDMARD, n (%)	66 (69.5)	48 (68.6)	18 (72.0)	0.947
Azathioprine, n (%)	-	-	-	-
Cyclophosphamide, n (%)	-	-	-	-
Cyclosporine, n (%)	2 (2.1)	2 (2.9)	-	-
Hydroxychloroquine sulfate, n (%)	32 (33.7)	25 (35.7)	7 (28.0)	0.650
Leflunomide, n (%)	19 (20.0)	16 (22.9)	3 (12.0)	0.382
Methotrexate, n (%)	29 (30.5)	20 (28.6)	9 (36.0)	0.660
Mycophenolate mofetil, n (%)	1 (1.1)	-	1 (4.0)	-
Sulfasalazine, n (%)	6 (6.3)	5 (7.1)	1 (4.0)	1.00
*RA disease duration*				
Mean days (SD)	3598.2 (3522.4)	3537.6 (3652.8)	3791.2 (3172.3)	0.804
Median days [IQR]	2633.0 [966.5–5518.5]	1533.0 [959.0–5612.0]	3230.0 [1008.3–5355.8]	0.477

DAS, disease activity score; DMARDs, disease-modifying antirheumatic drugs; csDMARDs: conventional synthetic DMARDs; IQR: interquartile range; n: number; RA: rheumatoid arthritis; and SD: standard deviation.

The median age observed at RA diagnosis was 54 [IQR = 41–60]. Overall, 91 discontinuations were identified (Supplementary Table S1), 67.0% had a DAS28TD0 and 59.3% DAS28TD1 (Supplementary Table S2). At TD0, 41.8% of discontinuations were associated with and in-target disease, while at TD1, the majority of discontinuations (40.7%) had no DAS28 recorded, and 35.2% reported an in-target RA (Supplementary Table S2).

**FIGURE 3:**
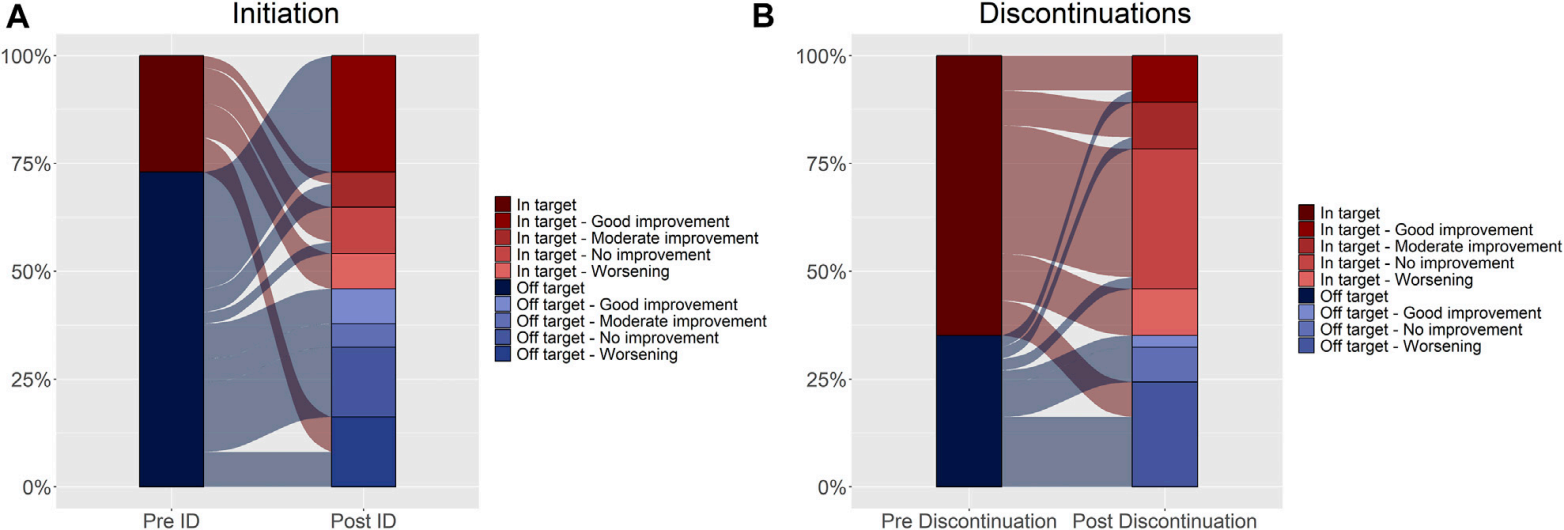
Disease activity trend assessed at initiation **(A)** and discontinuations **(B)**. Changes in DAS28 relative to the index date and discontinuations are shown in Figure 3 (Panel A, B, respectively). The reference unit is the subject in Panel A, and the discontinuation in Panel **(B)**. In the left column of both panels, disease activity expressed as DAS28 is reported and categorized as in-target (dark red) and off-target (dark blue) (pre-index date, Panel A and pre-discontinuation date, Panel **(B)**. In the right column, disease activity was further categorized based on the movement over time (post-index date, Panel A and post-discontinuation date, Panel **(B)** as good improvement, moderate improvement, no improvement, and worsening. Shades of red represent in-target conditions, and shades of blue represent off-target conditions. The flows connecting the different layers of the two bars represent the movement over time, relative to the index date (Panel A) and the discontinuation date (Panel B). The width of the flow reflects the proportion of subjects (Panel A) or discontinuations (Panel B).

Seventy patients (74.0%) had at least three DAS28 assessments recorded in the medical charts (Figure 2; Table 1). No statistical differences at the baseline were observed between these patients and those with less than three DAS28 values registered (Table 1). We identified 60 discontinuation events. Out of 70 patients with at least three DAS28 assessments available, 33 (47.1%) had at least one discontinuation; this distribution is similar to that observed in the general study population (47/95 patients, 49.5%) (Supplementary Table S1). Only six patients moved away from Tuscany, as reported in the medical charts, and no discontinuations of biologic treatment were detected before their moving. No significant differences were observed in the baseline characteristics between the population with at least one discontinuation and those without discontinuation (Supplementary Table S3). The DAS28T0 was available in 41 patients (58.6%) and the DAS28T1 in 66 (94.3%). At T0, 40% of patients were off-target and 41.4% had no DAS28 recorded, while at T1, 54.3% were in-target. At T1, patients with at least one discontinuation had a significant distribution of DAS28 associated with an off-target disease, while those continuing treatment displayed, most frequently, an in-target condition (Supplementary Table S4). Among the available 37 patients with both the DAS28T0 and DAS28T1 (Supplementary Table S5 and Table 2), 13 (35%) displayed a good improvement and six (16.2%) a moderate one (Table 2; Figure 3).

**TABLE 2:** Assessment of DAS28 at T0 and T1 within patients with at least three available DAS28.

Patients with both DAS28T0* and DAS28T1°	
Overall, n	37
DAS28T0* off-target^§^, n (%)	27 (73.0)
DAS28T0 off-target AND DAS28T1 off-target, n (%)	14 (52.0)
Good improvement^#^, n (%)	3 (21.4)
*Difference range*	[−4.4; −1.5]
Moderate improvement^+^, n (%)	3 (21.4)
*Difference range*	[−1.1; −0.6]
No improvement^, n (%)	5 (35.7)
*Difference range*	[-0.4; 0.0]
Worsening^£^, n (%)	3 (21.4)
*Difference range*	[0.1–0.9]
DAS28T0 off-target AND DAS28T1 in-target, n (%)	13 (48.0)
Good improvement, n (%)	10 (77.0)
*Difference range*	[−5.3; −1.5]
Moderate improvement, n (%)	2 (15.0)
*Difference range*	[−1.0; −0.7]
No improvement, n (%)	1 (8.0)
*Difference range*	[−0.5]
Worsening^£^, n (%)	-
*Difference range*	-
DAS28T0* in-target^ç^, n (%)	10 (27.0)
DAS28T0 in-target AND DAS28T1 off-target, n (%)	3 (30.0)
Worsening, n (%)	3 (100.0)
*Difference range*	[1.0; 2.7]
DAS28T0 in-target AND DAS28T1 in-target, n (%)	7 (70.0)
Good improvement, n (%)	-
*Difference range*	-
Moderate improvement, n (%)	1 (14.3)
*Difference range*	[-0.9]
No improvement, n (%)	3 (42.9)
*Difference range*	[-0.1; 0.0]
Worsening, n (%)	3 (42.9)
*Difference range*	[0.1; 0.8]

DAS, disease activity score; DMARDs, disease-modifying antirheumatic drugs; n, number; RA, rheumatoid arthritis.

* DAS28T0: the closest DAS28 value recorded before the index date, including the index date.

° DAS28T1: the closest DAS28 value recorded after the index date.

§ off-target: DAS28 > 3.2.

Ç in-target: DAS28 ≤ 3.2.

#Good improvement: difference > −1.2; range [-∞; -1.2].

+Moderate improvement: difference > −0.6; range [-1.2; -0.6].

^No improvement: difference ≤ -0.6; range [-0.6; 0].

£Worsening: difference >0 range (0; +∞].

The median time elapsed between the DAS28T0 assessment and index date was 36 days (IQR 0–132), while the median time between DAS28T1 assessment and index date was 93 days (IQR 31–252). The subjects with both pre- and post-index DAS28 measurements within 180 days of it were 22 (59.5%), and 20/22 had off-target disease (91%), while 2/22 (9%) had an in-target RA. Of these, five (22.7%) experienced a deterioration, with one (20.0%) moving from in-target to off-target, three (60.0%) remaining off-target, and one (20.0%) remaining in-target.

Most of the patients showed disease stability or improvement after initiating biologic DMARDs (75.7%) (Table 2; Figure 3), as well as after the discontinuation events (64.9%) (Table 3; Figure 3).

**TABLE 3:** Assessment of DAS28 in the discontinuation events, classified by chronological occurrence.

	Discontinuations available with both DAS28TD0* and DAS28TD1°			
	Overall	First event	Second event	Subsequent events
Patients, n	24	24	6	4
Events, n	37	24	6	7
*DAS28 assessments*				
DAS28TD0* off-target^§^, n (%)	13 (35.1)	9 (37.5)	2 (33.3)	2 (33.3)
DAS28TD0 off-target AND DAS28TD1° off-target, n (%)	10 (76.9)	7 (77.8)	2 (100.0)	1 (50.0)
Good improvement^#^, n (%)	1 (10.0)	1 (14.3)	-	-
*Difference range*	[ −3.0]	[-3.0]		
Moderate improvement^+^, n (%)	-	-	-	-
*Difference range*				
No improvement^, n (%)	3 (30.0)	2 (28.6)	1 (50.0)	
*Difference range*	[−0.5; 0.0]	[−0.5; 0.0]	[−0.1]	-[−0.1; 1.0]
Worsening^£^, n (%)	6 (0.6)	4 (57.1)	1 (50.0)	1 (100.0)
*Difference range*	[0.1; 2.6]	[0.1; 2.6]	[0.8]	[0.6]
DAS28TD0 off-target AND DAS28TD1 in-target, n (%)	3 (23.1)	2 (22.2)	-	1 (50.0)
Good improvement, n (%)	1 (33.3)	1 (50.0)	-	-
*Difference range*	[−2.9]	[−2.9]		
Moderate improvement, n (%)	2 (66.7)	1 (50.0)	-	1 (100.0)
*Difference range*	[−1.0; -0.6]	[−1.0]		[−0.6]
No improvement^, n (%)	-	-	-	-
*Difference range*				
Worsening, n (%)	-			
*Difference range*	-			
DAS28TD0* in-target, n (%)	24 (64.9)	15 (62.5)	4 (66.7)	5 (71.4)
DAS28TD0 in-target AND DAS28TD1 off-target, n (%)	3 (13.0)	2 (13.3)	1 (25.0)	-
Worsening, n (%)	3 (100.0)	2 (100.0)	1 (100.0)	-
*Difference range*	[1.7; 2.0]	[1.7; 2.0]	[1.8]	
DAS28TD0 in-target and DAS28TD1 in-target, n (%)	21 (87.5)	13 (86.7)	3 (75.0)	5 (100.0)
Good improvement, n (%)	3 (15.0)	1 (7.7)	1 (33.3)	1 (20.0)
*Difference range*	[−1.4; −1.3]	[−1.4]	[−1.3]	[−1.3]
Moderate improvement, n (%)	3 (15.0)	2 (15.4)	-	1 (20.0)
*Difference range*	[−1.1;−0.7]	[−1.1;−0.7]		[−0.8]
No improvement, n (%)	11 (52.4)	9 (69.2)	1 (33.3)	1 (20.0)
*Difference range*	[−0.3; 0.0]	[−0.3; 0.0]	[0.0]	[−0.2]
Worsening, n (%)	4 (20.0)	1 (7.7)	1 (33.3)	2 (40.0)
*Difference range*	[0.2; 0.7]	[0.7]	[0.4]	[0.2]

DAS, disease activity score and n, number.

* DAS28TD0: the closest DAS28 value recorded before the discontinuation date, including the discontinuation date.

° DAS28TD1: the closest DAS28 value recorded after the discontinuation date.

§ off-target: DAS28 > 3.2.

Ç in-target: DAS28 ≤ 3.2.

#Good improvement: difference > −1.2; range [-∞; -1.2].

+Moderate improvement: difference > −0.6; range [-1.2; -0.6].

^No improvement: difference ≤ -0.6; range [-0.6; 0].

^£^Worsening: difference >0 range (0; +∞].

Out of 33 patients with at least three DAS28 and a discontinuation, 24 had 37 events with both DAS28TD0 and DAS28TD1 (Supplementary Table S6). The reasons for discontinuations were deterioration of disease (13), adverse drug events (7), surgery/hospitalizations (5), pregnancy (4), and no information about were reported (8). Among these 37 discontinuation events (Supplementary Table S5 and Table 3), the disease activity showed a good or moderate improvement in 10 events (27.0%) (Table 3; Figure 3). The median time elapsed between the DAS28TD0 assessment and discontinuation date was 113 days (IQR 51–168), while the median time between the DAS28TD1 assessment and discontinuation date was 117 days (IQR 49–221). The subjects with both pre- and post-discontinuation DAS28 measurements within 180 days of it were 18 (48.6%), and 10/18 patients were in target and 8/10 off-target at TD0. Of these, nine (50.0%) experienced a deterioration, with one (11.1%) moving from in-target to off-target, six (66.7%) remaining off-target, and two (22.2%) remaining in-target.

Out of 24 patients with both DAS28TD0 and DAS28TD1, 11 had adverse events recorded. In particular, among the 37 discontinuations, we retrieved 15 adverse events, of which 9/15 occurred ±1 year at the discontinuation date and 3/9 showed off-target RA and disease worsening. These included neutropenia, bile acid increase, and pneumonia. Out of the six remaining adverse events reported within 1 year of the discontinuation, three (ovarian cancer, hypersensibility, and cough/sinusitis) occurred in a condition of the in-target disease and stability, one (intolerance) in an in-target and improvement disease, and one (hypersensibility) in an off-target and improvement condition. The drugs discontinued were etanercept, adalimumab, certolizumab, and abatacept. No further information was reported in the medical charts (Supplementary Table S7).

In sensitivity analysis, we almost confirmed the main analysis observations (Supplementary Table S8–S13).

## 4 Discussion

This study describing the relationship between drug utilization patterns assessed using HAD information and the disease activity reported in the medical charts of RA patients showed that over half of the discontinuation events had an in-target disease before and after the biologic interruption.

In line with the clinical recommendations (Smolen et al., 2014; Smolen et al., 2017) and literature evidence (Silvagni et al., 2018), in our study, etanercept and adalimumab were among the most frequently supplied index drugs. We found a high percentage of abatacept supplies in both the overall population and in the subgroup of patients with three available DAS28 assessments. A high number of DAS28 assessments could reflect closer monitoring of patients by rheumatologists, probably in relationship with the disease burden. At the time of observation, abatacept was recommended as a first-line biologic DMARD in subjects with co-morbidities (Smolen et al., 2010; Monti et al., 2017), and therefore its higher use in subjects with more assessments (i.e., those with more complicated disease) seems to be plausible. However, statistical significance was not confirmed; therefore, these results should be considered with caution.

When investigating the initiation of the first ever biologic drugs in subjects with available DAS28 measure before and after the ID, we observed that 73% of patients (27/37) started with an off-target disease that provides the rationale for prescribing a biologic drug in accordance with the clinical guidelines. However, (10/37) 27% of patients started biologic treatment with an in-target disease. It is important to remark that the causal relationship between disease activity and the initiation of biologics is conditioned by the temporal distance between the available DAS28 assessments and the initiation event. It is unlikely that a DAS28 measure recorded several years before and after the ID can provide a reliable disease activity measure at the time of biologic DMARD initiation. In this regard, it is important to note that patients with DAS28 assessed within 6 months of the ID (i.e., those with the most reliable disease activity assessment) showed off-target disease in the majority of cases (20/22 patients, 91%). In 13/27 patients starting with an off-target disease (48%), the disease control was achieved at the subsequent assessment, while in six (22.2%) users, the improvements were without achieving the disease control. These results are in line with those of other real-world studies. For instance, a study using the Corrona registry data and evaluating biologic naïve patients with moderate and severe RA pointed out that among 817 patients with severe RA and 779 with moderate disease, 41.2% and 60.1% achieved a controlled disease after 1 year, respectively (Kavanaugh et al., 2017).

When disease activity was measured in relationship with discontinuations, 24 (64.9%) events with both DAS28TD0 and DAS28TD1 measures available presented an in-target disease before discontinuing the treatment and 17/24 (70.8%) displayed disease improvement or stability after the discontinuation. Adverse events recorded in the medical charts rarely occurred in plausible temporal relationships to suggest a causal role for discontinuation events. These results suggest that, according to our discontinuation definition, disease control could often drive the clinical decision of tapering biologic DMARDs in accordance with the clinical guidelines (Smolen et al., 2014; Smolen et al., 2017) and disease remains controlled after tapering. The robustness of these findings is confirmed by the sensitivity analysis, performed to evaluate whether the discontinuation definition could have affected our observations. Even in this case, the time distance between available DAS28 assessments and the date of the discontinuation event could affect the reliability of the results (18/24 patients, 75%, had DAS28 assessments within 6 months before and after the discontinuation date). Overall, 10/18 patients were in target and 8/18 were off-target. Nine discontinuation events showed a disease worsening, six starting from an off-target condition, and four from an in-target disease. Out of these, only one patient with an off-target disease switched to a JAK inhibitor. In these cases, the decision to discontinue the treatment should have been driven by reasons other than the achievement of disease control [i.e., patient deterioration due to co-morbidity-related events (Listing et al., 2015), lack of biologic DMARD response (Olsen et al., 2019), safety issues (Capogrosso Sansone et al., 2015; Codreanu and Damjanov, 2015; Antonazzo et al., 2022), or non-clinical events] that deserve further investigations.

Our results about the in-target disease observed before discontinuations and disease improvement or stability after the interruption are in line with those in the medical literature. A prospective observational study on 43 first ever biologic DMARD users interrupting treatment showed that 58.1% of patients maintained discontinuation along with the in-target condition for up to 1 year. In these patients, the disease activity recorded after the biologic DMARD initiation was significantly lower than in patients restarting biologic DMARD, who relapsed to off-target within 1 year (Ochiai et al., 2021). Another multicenter observational study performed in Japan using data from medical records of 102 RA infliximab users having DAS28 < 3.2 for at least 24 weeks displayed that 55% of patients maintained disease stability and 43% achieved RA remission after infliximab discontinuation (Tanaka et al., 2010). In the HOPEFUL-3 study, a follow-up to the HOPEFUL-1 and HOPEFUL-2 studies evaluating adalimumab users in Japanese patients with early RA, out of 74 patients discontinuing adalimumab for low disease activity, 59 (79.7%) retained the status of low RA activity for about 4 years (Tanaka et al., 2017). Schlager et al. (2020), in a recent systematic literature review and meta-analysis of clinical trials and observational studies, highlighted that the low disease activity at the time of discontinuation should be explored as a predictor of interruption. However, this study displayed that among patients discontinuing biologic DMARDs owing to the low disease activity, the probability for RA relapsing was high at 13 months of observation (overall Odds Ratio, OR 3.87; 95% CI 2.31–6.49).

Our study has some elements of strength. First, we used ecological data from Tuscan HAD that have been consolidated from previous population-based studies (Gini et al., 2014; Convertino et al., 2021a; Convertino et al., 2021b; Convertino et al., 2021c; Trifirò et al., 2021; Convertino et al., 2023). Second, while disease activity is usually measured by proxies in HAD studies, in our investigation, instead, we used the medical charts that represent a more reliable source of this information. Third, since therapeutic indications of drugs are not recorded in the HAD in Italy, by linking information from medical charts, we could have the certainty that all biologic users included in our study had RA.

Some limitations have to be considered. First, the small number of patients included in the study could limit the extension of the results to the general population of RA patients. However, the majority of studies investigating similar topics in the literature are not more than ours (Tanaka et al., 2010; Tanaka et al., 2017; Schlager et al., 2020; Ochiai et al., 2021). Second, we have a limited number of disease activity assessments recorded in the medical charts, and this could affect our findings. Nevertheless, a UK study (Choy et al., 2012) showed that the baseline recording of DAS28 is available in about 45% starting a biologic DMARD therapy, which is lower than the frequency observed in our study (59%). The authors of this study hypothesized that disease activity is likely measured at any visit but recorded in medical charts more probably when a relevant improvement or worsening occurs. Therefore, the probability of not recording important disease activity modification in two consecutive measures, even distant in time, is unlikely. Third, the assessment of two consecutive DAS28 without time restriction could have influenced our results. However, the disease activity monitoring should be scheduled at least every 180 days (Smolen et al., 2010; Smolen et al., 2014; Smolen et al., 2017), which is not far from the 113 and 117 days in median observed before and after the discontinuation events in our study. Fourth, the quality of available data could have affected the results. However, we have carefully measured the characteristics of patients, and discontinuation events progressively dropped out from the analysis due to the lack of records of disease activities, and by comparing these with those remaining in the cohort, we controlled for the possible selection bias. Fifth, we cannot exclude that information bias (Grimes and Schulz, 2002) could have occurred. Sixth, the discontinuation measurement was based on drug coverage estimated through the DDD and not by the prescribed daily dose (PDD). Since the use of DDD could overestimate the persistence of patients and inversely underestimate the discontinuation assessment, we cannot exclude that a definition based on the actually prescribed dose could have changed by increasing the number and the timing of discontinuation events. However, we performed the sensitivity analysis by varying the discontinuation definition, which confirmed the robustness of the main analysis results.

## 5 Conclusion

In conclusion, half of the RA patients achieve an in-target disease after starting biologic DMARDs, and the disease activity trend mainly reveals RA stability or improvement. As far as discontinuations are concerned, the majority of discontinuation events reported an in-target condition before the interruption date, and disease control is also confirmed after the discontinuation. Future studies on a larger RA population are needed to confirm our findings and support the use of this discontinuation definition in Tuscan HAD as effectiveness proxy.

## Data Availability

The raw data supporting the conclusion of this article will be made available by the authors, without undue reservation.
