# Supplementary material for: Assessing disease activity of rheumatoid arthritis patients and drug-utilization patterns of biologic disease-modifying antirheumatic drugs in the Tuscany region, Italy
Source: Front Pharmacol. 2023 Sep 25;14:1244486. doi: 10.3389/fphar.2023.1244486 (PMC10561246; doi:10.3389/fphar.2023.1244486)
Supplement: Supplementary file 1 [file DataSheet1.pdf]

Supplementary Material

Assessing disease activity of rheumatoid arthritis patients and drug-utilization patterns of biologic disease-modifying anti-rheumatic drugs in Tuscany region, Italy

Irma Convertino[1†], Massimiliano Cazzato[2†], Silvia Tillati[3], Sabrina Giometto[3], Rosa Gini[4], Giulia Valdiserra[1], Emiliano Cappello[1], Sara Ferraro[1], Marco Bonaso[1], Claudia Bartolini[4], Olga Paoletti[4], Valentina Lorenzoni[5], Leopoldo Trieste[5], Matteo Filippi[6], Giuseppe Turchetti[5], Michele Cristofano[6], Corrado Blandizzi[1,7], Marta Mosca[2], Ersilia Lucenteforte[3*], Marco Tuccori[1,7]

[1] Unit of Pharmacology and Pharmacovigilance, Department of Clinical and Experimental Medicine, University of Pisa, Pisa, Italy
[2] Unit of Rheumatology, University Hospital of Pisa, Pisa, Italy
[3] Unit of Medical Statistics, Department of Clinical and Experimental Medicine, University of Pisa, Pisa, Italy
[4] Tuscan Regional Healthcare Agency, Florence, Italy
[5] Institute of Management, Scuola Superiore Sant'Anna, Pisa, Italy
[6] Direzione Medica di Presidio, University Hospital of Pisa
[7] Unit of Adverse Drug Reactions Monitoring, University Hospital of Pisa, Pisa, Italy

Irma Convertino[1†] and Massimiliano Cazzato[2†] contributed equally to this work and share first authorship

*** Correspondence:**
Corresponding Author: Ersilia Lucenteforte, Associate Professor of Medical Statistics, Unit of Medical Statistics, Department of Clinical and Experimental Medicine, University of Pisa, Via Roma 67, 56126 Pisa; E-mail: ersilia.lucenteforte@unipi.it

Supplementary Figure S1 Flow of data (decryption process) **2**

Supplementary Figure S2 Disease duration: A Categories of disease duration (days); B Duration per single patient **3**

Supplementary Table S1 Distribution of discontinuations in the included patients **4**

Supplementary Table S2 Disease activity (DAS28) at discontinuation **5**

Supplementary Table S3 Baseline characteristics of RA first ever biologic DMARDs users having at least 3 DAS28 available **6**

Supplementary Table S4 Description of DAS28 in patients with at least 3 DAS28 assessments **7**

Supplementary Table S5 Baseline characteristics of patients with both DAS28T0 and DAS28T1 and those without **8**

Supplementary Table S6 Baseline characteristics of patients with both DAS28TD0 and DAS28TD1 and those without **9**

Supplementary Table S7 Description of the adverse events recorded in the medical charts **10**

Supplementary Table S8 Distribution of discontinuations in the sensitivity analysis **11**

Supplementary Table S9 Baseline characteristics of patients having at least 3 DAS28 assessments available (sensitivity analysis)..**12**

Supplementary Table S10 Distribution of DAS28 in patients with at least 3 DAS28 assessments (sensitivity analysis) **13**

Supplementary Table S11 Disease activity (DAS28) at discontinuation (sensitivity analysis)....................**14**

Supplementary Table S12 Baseline characteristics of patients with both DAS28TD0 and DAS28TD1 and those without (sensitivity analysis)..**15**

Supplementary Table S13 Assessment of DAS28 in the discontinuations, classified by chronological occurrence (sensitivity analysis) **16**

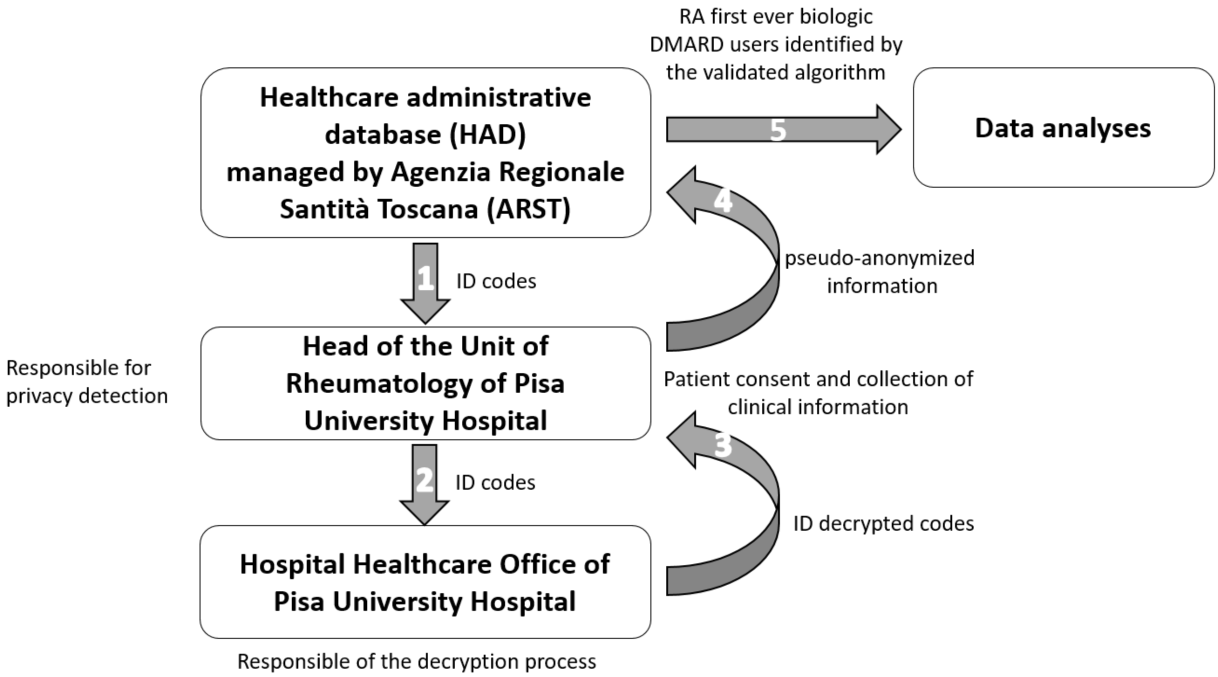

Supplementary Figure S1 Flow of data (decryption process)

Data were extracted by the Agenzia Regionale di Sanità Toscana (ARST) from the HAD. 1 The file with the unique anonymous identification code (ID) was sent to the head of the Rheumatology Unit of the Pisa University Hospital (responsible of privacy). 2 The head of healthcare office of the Pisa University Hospital decrypted patient ID codes. 3 The decrypted codes returned to the Rheumatology head who collected patient consent and related data from medical charts. 4 The pseudo-anonymized information returned to the ARST. 5 The ARST linked data of the extracted patients to those collected from the corresponding medical charts.

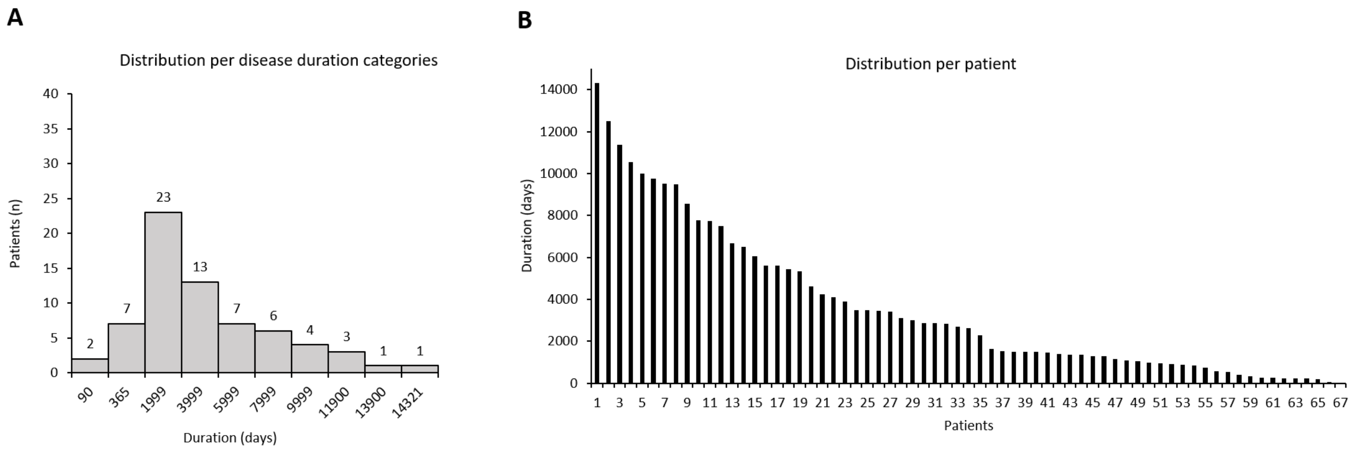

Supplementary Figure S2 Disease duration: A Categories of disease duration (days); B Duration per single patient

The disease duration was measured as the time run from the date of the first rheumatoid arthritis (RA) diagnosis recorded in the healthcare administrative database (HAD) to the date of the first biologic DMARD supply recorded in the HAD, i.e. the index date (ID). Section A shows the distribution of patients according to categories of disease duration quantified in days; Section B points out the distribution of disease duration per single patient.

Supplementary Table S1 Distribution of discontinuations in the included patients

	**RA first ever biologic DMARD users with DAS28 assessments**			***p-value***
	**Overall**	**With at least 3 values**	**With < 3 values**	
Patients, n	95	70	25	
Discontinuations				
With at least one discontinuation, n (%)	47 (49.5)	33 (47.1)	14 (56.0)	0.598
Categories (according to distribution)				
0	48 (50.5)	37 (52.9)	11 (44.0)	0.816
1	26 (27.4)	19 (27.1)	7 (28.0)	
2 - 3	14 (14.7)	10 (14.3)	4 (16.0)	
≥4	7 (7.4)	4 (5.7)	3 (12.0)	
Discontinuations, n	91	60	31	
mean (SD)	0.95 (1.3)	0.8 (1.2)	1.2 (1.6)	0.229
median [IQR]	0 [0-1]	0 [0-1]	1 [0-2]	0.592

DAS: Disease activity score; DMARDs: disease modifying anti-rheumatic drugs; RA: rheumatoid arthritis; IQR: interquartile range; n: number; SD: standard deviation

Supplementary Table S2 Disease activity (DAS28) at discontinuation

	**Overall**
Discontinuations, n	91
DAS28TD0 assessments*	
Discontinuations with DAS28TD0*, n (%)	61 (67.0)
Mean DAS28TD0* (SD)	3.3 (1.4)
Median DAS28TD0* [IQR]	3.1 [2.1-4.2]
Categories (according to target)	
Discontinuations with DAS28TD0* off-target, n (%)	23 (25.2)
Discontinuations with DAS28TD0* in-target, n (%)	38 (41.8)
Discontinuations without DAS28TD0*, n (%)	30 (33.0)
DAS28TD1° assessments	
Discontinuations with DAS28TD1°, n (%)	54 (59.3)
Mean DAS28TD1° (SD)	3.1 (1.5)
Median DAS28TD1° [IQR]	2.3 [1.9-4.2]
Categories (according to target)	
Discontinuations with DAS28TD1° off-target, n (%)	22 (24.2)
Discontinuations with DAS28TD1° in-target, n (%)	32 (35.2)
Number of discontinuations without DAS28TD1°, n (%)	37 (40.7)
Difference between DAS28TD0 and DAS28TD1°*	
Discontinuations with DAS28TD0* and DAS28TD1°, n (%)	37 (40.7)
mean (SD)	-0.09 (1.1)
median [IQR]	0.0 [-0.6-0.2]
Categories (according to improvement)	
Good improvement#, n (%)	5 (5.4)
Moderate improvement+, n (%)	5 (5.4)
No improvement^, n (%)	14 (15.4)
Worsening£, n (%)	13 (14.3)
Not available	54 (59.3)
*Days from DAS28TD1° to DAS28TD0**	
mean (SD)	344.4 (320.9)
median [IQR]	259.0 [171.0-385.0]

DAS: Disease activity score; IQR: interquartile range; n: number; SD: standard deviation

* DAS28TD0: closest DAS28 value recorded before the discontinuation date, including the discontinuation date

° DAS28TD1: closest DAS28 value recorded after the discontinuation date

§ off-target: DAS28 > 3.2

Ç in-target: DAS28 ≤ 3.2

#Good improvement: difference > -1.2; range [-∞; -1.2)

+Moderate improvement: difference > -0.6; range [-1.2; -0.6)

^No improvement: difference ≤-0.6; range [-0.6; 0]

£Worsening: difference > 0 range (0; +∞]

Supplementary Table S3 Baseline characteristics of RA first ever biologic DMARDs users having at least 3 DAS28 available

	RA first ever biologic DMARD users with at least 3 DAS28 assessments			*p-value*
	**Overall**	**With at least one discontinuation**	**Without discontinuations**	
Patients, n	70 (100.0)	33 (47.1)	37 (52.9)	
Females, n (%)	55 (78.6)	26 (78.8)	29 (78.4)	0.328
Age, mean (SD)	59.3 (12.4)	57.1 (11.0)	61.6 (13.4)	0.141
Index biologic DMARDs				
Adalimumab, n (%)	11 (15.7)	6 (18.2)	5 (13.5)	0.836
Certolizumab pegol, n (%)	4 (5.7)	1 (3.0)	3 (8.1)	0.691
Etanercept, n (%)	20 (28.6)	6 (18.2)	14 (37.8)	0.121
Golimumab, n (%)	4 (5.7)	2 (6.1)	2 (5.4)	0.691
Infliximab, n (%)	1 (1.4)	1 (3.0)	-	-
Abatacept, n (%)	28 (40.0)	16 (48.5)	12 (32.4)	0.261
Tocilizumab, n (%)	2 (2.9)	1 (3.0)	1 (2.7)	0.524
csDMARDs				
At least one csDMARD, n (%)	48 (68.6)	23 (69.7)	25 (67.6)	1.00
Azathioprine, n (%)	-	-	-	-
Cyclophosphamide, n (%)	-	-	-	-
Cyclosporine, n (%)	2 (2.9)	-	2 (5.4)	-
Hydroxychloroquine sulfate, n (%)	25 (35.7)	13 (39.4)	12 (32.4)	0.721
Leflunomide, n (%)	16 (22.9)	6 (18.2)	10 (27.0)	0.552
Methotrexate, n (%)	20 (28.6)	10 (30.3)	10 (27.0)	0.970
Mycophenolate mofetil, n (%)	-	-	-	-
Sulfasalazine, n (%)	5 (7.1)	1 (3.0)	4 (10.8)	0.361
RA disease duration				
mean days (SD)	3537.6 (3652.8)	3857.3 (3672.1)	3230.2 (3679.8)	0.545
median days [IQR]	1533.0 [5612-959]	2710.0 [1300.0-5436.0]	1421.0 [923.0-5266.0]	0.473

DAS: Disease activity score; DMARDs: disease modifying anti-rheumatic drugs; csDMARDs: conventional synthetic DMARDs; IQR: interquartile range; n: number; RA: rheumatoid arthritis; SD: standard deviation

Supplementary Table S4 Description of DAS28 in patients with at least 3 DAS28 assessments

Patients	RA first ever biologic DMARD users with at least 3 DAS28 assessments			*p-value*
	**Overall**	**With at least one discontinuation**	**Without discontinuations**	
Number (%)	70 (100.0)	33 (47.1)	37 (52.9)	
DAS28T0 assessments*				
With DAS28T0, n (%)	41 (58.6)	21 (63.6)	20 (54.1)	0.569
Categories (according to target) at T0				
Off-target§, n (%)	28 (40.0)	15 (45.4)	13 (35.1)	
In-targetÇ, n (%)	13 (18.6)	6 (18.2)	7 (18.9)	0.652
Without DAS28T0, n (%)	29 (41.4)	12 (36.4)	17 (45.9)	
DAS28T1° assessments				
With DAS28T1, n (%)	66 (94.3)	31 (93.9)	35 (94.6)	0.691
Categories (according to target) at T1				
Off-target§, n (%)	28 (40.0)	18 (54.5)	10 (27.0)	
In-targetÇ, n (%)	38 (54.3)	13 (39.4)	25 (67.6)	0.053
Without DAS28T1, n (%)	4 (5.7)	2 (6.1)	2 (5.4)	
Difference between DAS28T0 and DAS28T1°*				
Number of patients, n (%)	37 (52.9)	19 (57.6)	18 (48.6)	0.612
mean (SD)	-0.9 (1.7)	-0.8 (1.6)	-1.1 (1.8)	0.584
median [IQR]	-0.6 [(-1.8)]-0.0	-0.3 [-1.7-0.0]	-0.8 [-1.9-0.1]	0.612
Categories (according to improvement)				*0.792*
Good improvement#, n (%)	13 (18.6)	6 (18.2)	7 (18.9)	
Moderate improvement+, n (%)	6 (8.6)	3 (9.1)	3 (8.1)	
No improvement^, n (%)	9 (12.9)	6 (18.2)	3 (8.1)	
Worsening£, n (%)	9 (12.9)	4 (12.1)	5 (13.5)	
Not available, n (%)	33 (47.1)	14 (42.4)	19 (51.4)	

DAS: Disease activity score; DMARDs: disease modifying anti-rheumatic drugs; IQR: interquartile range; n: number; RA: rheumatoid arthritis; SD: standard deviation
* DAS28T0: closest DAS28 value recorded before the index date, including the index date
° DAS28T1: closest DAS28 value recorded after the index date
§ off-target: DAS28 > 3.2
Ç in-target: DAS28 ≤ 3.2
#Good improvement: difference > -1.2; range [-∞; -1.2)
+Moderate improvement: difference > -0.6; range [-1.2; -0.6)
^No improvement: difference ≤-0.6; range [-0.6; 0]
£Worsening: difference > 0 range (0; +∞]

Supplementary Table S5 Baseline characteristics of patients with both DAS28T0 and DAS28T1 and those without

	**RA first ever biologic DMARD users with at least 3 DAS28 assessments**		
	**With both DAS28T0* and DAS28T1°**	**Without DAS28T0* and DAS28T1***	***p-value***
Patients, n	37	33	
Females, n (%)	28 (84.8)	27 (84.4)	0.45
Age, mean (SD)	59.6 (12.3)	59.0 (12.6)	0.85
Index biologic DMARDs			0.76
Adalimumab, n (%)	6 (16.2)	5 (15.2)	
Certolizumab pegol, n (%)	2 (5.4)	2 (6.0)	
Etanercept, n (%)	9 (24.3)	11 (33.3)	
Golimumab, n (%)	3 (8.1)	1 (3.0)	
Infliximab, n (%)	-	1 (3.0)	
Abatacept, n (%)	16 (43.2)	12 (36.4)	
Tocilizumab, n (%)	1 (2.7)	1 (3.0)	
csDMARDs			
At least one csDMARD, n (%)	27 (73.0)	21 (63.6)	0.56
Azathioprine, n (%)	-	-	-
Cyclophosphamide, n (%)	-	-	-
Cyclosporine, n (%)	2 (5.4)	-	-
Hydroxychloroquine sulfate, n (%)	15 (40.5)	10 (30.3)	0.52
Leflunomide, n (%)	9 (24.3)	7 (21.2)	0.98
Methotrexate, n (%)	12 (32.4)	8 (24.2)	0.62
Mycophenolate mofetil, n (%)	-	-	
Sulfasalazine, n (%)	2 (5.4)	3 (9.1)	0.66

DAS: Disease activity score; DMARDs: disease modifying anti-rheumatic drugs; csDMARDs: conventional synthetic DMARDs; IQR: interquartile range; n: number; RA: rheumatoid arthritis; SD: standard deviation
* DAS28T0: closest DAS28 value recorded before the index date, including the index date
° DAS28T1: closest DAS28 value recorded after the index date

Supplementary Table S6 Baseline characteristics of patients with both DAS28TD0 and DAS28TD1 and those without

	RA first ever biologic DMARD users with at least 3 DAS28 assessments and at least one discontinuation		*p-value*
	**With both DAS28TD0* and DAS28TD1° available**	**Without both DAS28TD0* and DAS28TD1° available**	
Patients, n	24	9	
Females, n (%)	18 (75.0)	8 (88.9)	0.64
Age, mean (SD)	56.9 (12.0)	57.7 (8.5)	0.86
Index biologic DMARDs			0.95
Adalimumab, n (%)	4 (16.7)	2 (22.2)	
Certolizumab pegol, n (%)	1 (4.2)	-	
Etanercept, n (%)	5 (20.8)	1 (11.1)	
Golimumab, n (%)	1 (4.2)	1 (11.1)	
Infliximab, n (%)	1 (4.2)	-	
Abatacept, n (%)	11 (45.8)	5 (55.6)	
Tocilizumab, n (%)	1 (4.2)	-	
csDMARDs			
At least one csDMARD, n (%)	18 (75.0)	5 (55.6)	0.40
Azathioprine, n (%)	-	-	-
Cyclophosphamide, n (%)	-	-	-
Cyclosporine, n (%)	-	-	-
Hydroxychloroquine sulfate, n (%)	8 (33.3)	5 (55.6)	0.43
Leflunomide, n (%)	5 (20.8)	1 (11.1)	1.00
Methotrexate, n (%)	10 (41.7)	-	-
Mycophenolate mofetil, n (%)	-	-	-
Sulfasalazine, n (%)	1 (4.2)	-	-

DAS: Disease activity score; DMARDs: disease modifying anti-rheumatic drugs; csDMARDs: conventional synthetic DMARDs; n: number; RA: rheumatoid arthritis; SD: standard deviation

* DAS28TD0: closest DAS28 value recorded before the discontinuation date, including the discontinuation date

° DAS28TD1: closest DAS28 value recorded after the discontinuation date

Supplementary Table S7 Description of the adverse events recorded in the medical charts

Event description*	Time at discontinuation (+/- days)§	Drug discontinued	Disease activity	Disease trend
lack of therapeutic effect	-5968	abatacept	off-target	worsening
hypersensibility	-1390	certolizumab	in-target	stability
lung disease	-800	adalimumab	in-target	stability
increased transaminases	-683	abatacept	off-target	worsening
hepatitis	-621	certolizumab	off-target	worsening
ovarian cancer	**-98**	etanercept	in-target	stability
hypersensibility	**-82**	adalimumab	in-target	stability
neutropenia	**-82**	adalimumab	**off-target**	**worsening**
bile acids' increase	**-17**	certolizumab	**off-target**	**worsening**
cough / sinusitis	**+13**	adalimumab	in-target	stability
hypersensibility	**+116**	etanercept	**off-target**	improvement
pneumonia	**+160**	certolizumab	**off-target**	**worsening**
intolerance	**+231**	abatacept	in-target	improvement
nausea	+469	etanercept	off-target	improvement
lack of therapeutic effect	+949	tocilizumab	in-target	stability

* Adverse events recorded in the medical charts of the 24 patients with DAS28 available before and after the discontinuation

§ Time lasting between the date of the event recorded in the medical charts and that of the discontinuation assessed through the healthcare administrative database

Supplementary Table S8 Distribution of discontinuations in the sensitivity analysis

	RA first ever biologic DMARD users with DAS28 assessments			*p-value*
	**Overall**	**With at least 3 values**	**With < 3 values**	
Patients, n	95	70	25	
Discontinuations				
With at least one discontinuation, n (%)	72 (75.8)	53 (75.7)	19 (76)	0.808
Categories (according to distribution), n (%)				
0	23 (24.2)	17 (24.3)	6 (24.0)	0.972
1	14 (14.7)	10 (14.3)	4 (16.0)	
2 - 3	32 (33.7)	23 (32.9)	9 (36.0)	
≥4	26 (27.4)	20 (28.6)	6 (24.0)	
Discontinuations, n	256	190	66	
mean (SD)	2.7 (2.6)	2.7 (2.5)	2.6 (2.7)	0.902
median [IQR]	2 [1-4]	2 [0.75-4]	2 [0.5-3.5]	0.833

DAS: Disease activity score; DMARDs: disease modifying anti-rheumatic drugs; RA: rheumatoid arthritis; IQR: interquartile range; n: number; SD: standard deviation

Supplementary Table S9 Baseline characteristics of patients having at least 3 DAS28 assessments available (sensitivity analysis)

	RA first ever biologic DMARD users with at least 3 DAS28 assessments			*p-value*
	**Overall**	**With at least one discontinuation**	**Without discontinuations**	
Patients, n (%)	70 (100.0)	53 (75.7)	17 (24.3)	
Females, n (%)	55 (78.5)	44 (83.0)	11 (64.7)	0.759
Age, mean (SD)	59.3 (12.4)	58.8 (12.3)	61.6 (13)	0.488
Index biologic DMARDs				0.331
Adalimumab, n (%)	11 (15.7)	7 (13.2)	4 (23.5)	
Certolizumab pegol, n (%)	4 (5.7)	2 (3.8)	2 (11.8)	
Etanercept, n (%)	20 (28.6)	15 (28.3)	5 (29.4)	
Golimumab, n (%)	4 (5.7)	3 (5.7)	1 (5.9)	
Infliximab, n (%)	1 (1.4)	1 (1.9)	-	
Abatacept, n (%)	28 (40.0)	24 (45.3)	4 (23.5)	
Tocilizumab, n (%)	2 (2.9)	1 (1.9)	1 (5.9)	
csDMARDs				
At least one csDMARD, n (%)	48 (68.6)	40 (75.5)	8 (47.1)	0.058
Azathioprine, n (%)	-	-	-	-
Cyclophosphamide, n (%)	-	-	-	-
Cyclosporine, n (%)	2 (1.9)	2 (3.8)	-	-
Hydroxychloroquine sulfate, n (%)	40 (37.4)	22 (41.5)	3 (17.6)	0.135
Leflunomide, n (%)	24 (22.4)	15 (28.3)	1 (5.9)	0.094
Methotrexate, n (%)	36 (33.6)	14 (26.4)	6 (35.3)	0.543
Mycophenolate mofetil, n (%)	-	-	-	-
Sulfasalazine, n (%)	5 (4.7)	4 (7.5)	1 (5.9)	1.00
RA disease duration				
mean days (SD)	3537.6 (3652.8)	3191.2 (3285.8)	4957.9(4832.7)	0.173
median days [IQR]	1533 [5612-959]	1496 [5384-935]	3430 [8787.25-844]	0.474

DAS: Disease activity score; DMARDs: disease modifying anti-rheumatic drugs; csDMARDs: conventional synthetic DMARDs; IQR: interquartile range; n: number; RA: rheumatoid arthritis; SD: standard deviation

Supplementary Table S10 Distribution of DAS28 in patients with at least 3 DAS28 assessments (sensitivity analysis)

**Patients**	**RA first ever biologic DMARD users with at least 3 DAS28 assessments**			***p-value***
	**Overall**	**With at least one discontinuation**	**Without discontinuations**	
Number	70	53	17	
DAS28T0 assessments*				
With DAS28T0, n (%)	41 (58.6)	33 (62.3)	8 (47.1)	0.410
Categories (according to target) at T0				
Off-target[§], n (%)	28 (40.0)	24 (45.3)	4 (23.5)	
In-target[ç], n (%)	13 (18.6)	9 (17.0)	4 (23.5)	0.281
Without DAS28T0, n (%)	29 (41.4)	20 (37.7)	9 (52.9)	
DAS28T1° assessments				
With DAS28T1, n (%)	66 (94.3)	50 (94.3)	16 (94.1)	0.571
Categories (according to target) at T1				
Off-target[§], n (%)	28 (40.0)	23 (43.4)	5 (29.4)	
In-target[ç], n (%)	38 (54.3)	27 (50.9)	11 (64.7)	0.583
Without DAS28T1, n (%)	4 (5.7)	3 (5.7)	1 (5.9)	
Difference between DAS28T0 and DAS28T1°*				
Number of patients, n (%)	37 (52.9)	30 (56.6)	7 (41.2)	0.407
mean (SD)	-0.9 (1.7)	-1.1 (1.7)	-0.07 (1.2)	0.137
median [IQR]	-0.6 [-1.8-0.0]	-0.8 [-2.1-0.0]	0.1 [-0.7-0.6]	0.242
Categories (according to improvement)				0.198
Good improvement[#], n (%)	13 (18.6)	12 (22.6)	1 (5.9)	
Moderate improvement[+], n (%)	6 (8.6)	5 (9.4)	1 (5.9)	
No improvement^, n (%)	9 (12.9)	8 (15.1)	1 (5.9)	
Worsening[£], n (%)	9 (12.9)	5 (9.4)	4 (23.5)	
Not available, n (%)	33 (47.1)	23 (43.4)	10 (58.8)	

DAS: Disease activity score; DMARDs: disease modifying anti-rheumatic drugs; IQR: interquartile range; n: number; RA: rheumatoid arthritis; SD: standard deviation

* DAS28T0: closest DAS28 value recorded before the index date, including the index date

° DAS28T1: closest DAS28 value recorded after the index date

§ off-target: DAS28 > 3.2

Ç in-target: DAS28 ≤ 3.2

#Good improvement: difference > -1.2; range [-∞; -1.2)

[+]Moderate improvement: difference > -0.6; range [-1.2; -0.6)

^No improvement: difference ≤-0.6; range [-0.6; 0]

[£]Worsening: difference > 0 range (0; +∞]

Supplementary Table S11 Disease activity (DAS28) at discontinuation (sensitivity analysis**)**

	**Overall**
Discontinuations, n	256
DAS28TD0 assessments*	
Discontinuations with DAS28TD0*, n (%)	180 (70.3)
Mean DAS28TD0* (SD)	3.03 (1.26)
Median DAS28TD0* [IQR]	2.6 [2.1-3.7]
Categories (according to target)	
Discontinuations with DAS28TD0* off-target, n (%)	55 (21.5)
Discontinuations with DAS28TD0* in-target, n (%)	125 (48.8)
Discontinuations without DAS28TD0*, n (%)	76 (29.7)
DAS28TD1° assessments	
Discontinuations with DAS28TD1°, n (%)	165 (64.5)
Mean DAS28TD1° (SD)	2.75 (1.23)
Median DAS28TD1° [IQR]	2.3 [1.9-3.4]
Categories (according to target)	
Discontinuations with DAS28TD1° off-target, n (%)	44 (17.2)
Discontinuations with DAS28TD1° in-target, n (%)	121 (47.3)
Number of discontinuations without DAS28TD1°, n (%)	91 (35.5)
Difference between DAS28TD0 and DAS28TD1°*	
Discontinuations with DAS28TD0* and DAS28TD1°, n (%)	127 (49.6)
mean (SD)	-0.26 (1.14)
median [IQR]	-0.1 [-0.7-0.2]
Categories (according to improvement)	
Good improvement#, n (%)	19 (7.4)
Moderate improvement+, n (%)	16 (6.3)
No improvement^, n (%)	55 (21.5)
Worsening£, n (%)	37 (14.5)
Not available	129 (50.4)
*Days from DAS28TD1° to DAS28TD0**	
mean (SD)	373.7 (294.2)
median [IQR]	290.0 [183.0-414.0]

DAS: Disease activity score; IQR: interquartile range; n: number; SD: standard deviation

* DAS28TD0: closest DAS28 value recorded before the discontinuation date, including the discontinuation date

° DAS28TD1: closest DAS28 value recorded after the discontinuation date

§ off-target: DAS28 > 3.2

Ç in-target: DAS28 ≤ 3.2

#Good improvement: difference > -1.2; range [-∞; -1.2)

+Moderate improvement: difference > -0.6; range [-1.2; -0.6)

^No improvement: difference ≤-0.6; range [-0.6; 0]

£Worsening: difference > 0 range (0; +∞]

Supplementary Table S12 Baseline characteristics of patients with both DAS28TD0 and DAS28TD1 and those without (sensitivity analysis)

	**RA first ever biologic DMARD users with at least 3 DAS28 assessments and at least one discontinuation**		***p-value***
	**With both DAS28TD0* and DAS28TD1° available**	**Without both DAS28TD0* and DAS28TD1° available**	
Patients, n	42	11	
Females, n (%)	33 (78.6)	9 (81.8)	1.00
Age, mean (SD)	58.5 (13.2)	58.5 (9.6)	0.70
Index biologic DMARDs			0.182
Adalimumab, n (%)	6 (14.3)	2 (18.2)	
Certolizumab pegol, n (%)	2 (4.8)	1 (9.1)	
Etanercept, n (%)	12 (28.6)	3 (27.3)	
Golimumab, n (%)	1 (2.4)	2 (18.2)	
Infliximab, n (%)	1 (2.4)	-	
Abatacept, n (%)	19 (45.2)	2 (18.2)	
Tocilizumab, n (%)	1 (2.4)	1 (9.1)	
csDMARDs			
At least one csDMARD, n (%)	32 (76.2)	7 (63.6)	0.45
Azathioprine, n (%)	-	-	-
Cyclophosphamide, n (%)	-	-	-
Cyclosporine, n (%)	2 (4.8)	-	-
Hydroxychloroquine sulfate, n (%)	16 (38.1)	2 (18.2)	0.30
Leflunomide, n (%)	13 (31.0)	1 (9.1)	0.25
Methotrexate, n (%)	12 (28.6)	5 (45.5)	0.30
Mycophenolate mofetil, n (%)	-	1 (9.1)	-
Sulfasalazine, n (%)	3 (7.1)	-	-

DAS: Disease activity score; DMARDs: disease modifying anti-rheumatic drugs; csDMARDs: conventional synthetic DMARDs; n: number; RA: rheumatoid arthritis; SD: standard deviation

* DAS28TD0: closest DAS28 value recorded before the discontinuation date, including the discontinuation date

° DAS28TD1: closest DAS28 value recorded after the discontinuation date

Supplementary Table S13 Assessment of DAS28 in the discontinuations, classified by chronological occurrence (sensitivity analysis)

	Discontinuations with both DAS28TD0* and DAS28TD1° available			
	**Overall**	**First event**	**Second event**	**Subsequent events**
Patients, n	42	42	33	21
Events, n	127	42	33	52
DAS28 assessments				
DAS28TD0* off-target[§], n (%)	37 (29.1)	17 (40.5)	6 (18.2)	14 (26.9)
DAS28TD0 off-target AND DAS28TD1° off-target, n (%)	18 (48.6)	10 (58.8)	2 (33.3)	6 (42.9)
Good improvement[#], n (%)	3 (16.7)	3 (30.0)	-	-
Difference range	[-3.0;-2.0]	[-3.0;-2.0]		
Moderate improvement[+], n (%)	2 (11.1)	2 (20.0)	-	-
Difference range	[-1.1;-0.9]	[-1.1-(-0.9)]		
No improvement^, n (%)	5 (27.8)	-2 (20.0)	1 (50.0)	2 (33.3)
Difference range	[-0.1;0.0]	[0.0]	[-0.1]	[-0.1]
Worsening°, n (%)	8 (44.4)	3 (30.0)	1 (50.0)	4 (66.7)
Difference range	[0.1-2.6]	0.1-2.6	[0.2]	[0.3-0.8]
DAS28TD0 off-target AND DAS28TD1 in-target, n (%)	19 (51.4)	7 (41.2)	4 (66.7)	8 (57.1)
Good improvement, n (%)	12 (63.1)	7 (100.0)	2 (50.0)	3 (37.5)
Difference range	[-5.3;-1.5]	[-5.3;-1.5]	[-2.9;-1.7]	[-2.4;-2.3]
Moderate improvement, n (%)	4 (21.1)	-	-	4 (50.0)
Difference range	[-1.0;-0.9]			[-1.0;-0.9]
No improvement, n (%)	3 (15.8)	-	2 (50.0)	1 (12.5)
Difference range	[-0.6;-0.3]		[-0.4;-0.3]	[-0.6]
Worsening°, n (%)	-	-	-	-
Difference range				
DAS28TD0* in-target[Ç], n (%)	90 (70.9)	25 (59.5)	27 (81.8)	38 (73.1)
DAS28TD0 in-target AND DAS28TD1 off-target, n (%)	9 (10.0)	2 (8.0)	5 (18.5)	2 (5.3)
DAS28TD0 in-target AND DAS28TD1 in-target, n (%)	81 (90.0)	23 (92.0)	22 (81.5)	36 (94.7)
Good improvement, n (%)	4 (4.9)	-	1 (4.5)	3 (8.3)
Difference range	[-1.4;-1.3]		[-1.4]	[-1.4;-1.3]
Moderate improvement, n (%)	10 (12.3)	4 (17.4)	2 (9.1)	4 (11.1)
Difference range	[-1.1;-0.6]	[-1.1;-0.7]	[-1.1;-0.7]	[-1.0;-0.6]
No improvement, n (%)	47 (58.0)	17 (73.9)	14 (63.6)	16 (44.4)
Difference range	[-0.6;0.0]	[-0.6;0.0]	[-0.5;0.0]	[-0.4;0.0]
Worsening°, n (%)	20 (24.7)	2 (8.7)	5 (22.7)	13 (36.1)
Difference range	[0.1;1.2]	[0.5;1.1]	[0.4;1.2]	[0.1:1.2]

DAS: Disease activity score; n: number
* DAS28TD0: closest DAS28 value recorded before the discontinuation date, including the discontinuation date
° DAS28TD1: closest DAS28 value recorded after the discontinuation date
§ off-target: DAS28 > 3.2
Ç in-target: DAS28 ≤ 3.2
#Good improvement: difference > -1.2; range [-∞; -1.2)
[+]Moderate improvement: difference ≥ -0.6; range [-1.2; -0.6)
^No improvement: difference ≤-0.6; range [-0.6; 0]
° Worsening: difference > 0 range (0; +∞]
